# Management of acute bronchospasm respiratory distress with CPAP ventilation associated with nebulization in the prehospital emergency setting

**DOI:** 10.1186/cc10741

**Published:** 2012-03-20

**Authors:** J Cuny, C Berteloot, P Goldstein, E Wiel

**Affiliations:** 1 CHRU de Lille, France

## Introduction

In emergency medicine, noninvasive ventilation (NIV) has grown up for COPD and acute pulmonary edema through the use of continuous positive airway pressure (CPAP). Recently, several studies have reported the use of NIV coupled with nebulized bronchodilators to optimize the management of acute asthma patients in emergency departments and ICUs. This has indicated an improvement in gas exchanges, decreased lung resistances and decreased work of breathing. The purpose of this study is to assess prehospital practices in CPAP for these patients, to target patients for its use, and to compare clinical data before and after achievement of CPAP with nebulization.

## Methods

We have conducted a retrospective, descriptive and observational study, by collecting all files (EMA, Dispatching Center) for each patient receiving CPAP associated with nebulization, for pulmonary bronchospasm (excluding acute pulmonary edema), and supported by the emergency medical service. Several data were analyzed: age, sex, history, severity signs, cardiac and respiratory rate, blood pressure, pulse oxymetry, need for intubation, nebulization of β_2_-agonists, anticholinergics, intravenous corticosteroids, and arterial blood gases.

## Results

Over an 18-month period, 21 patients were enrolled: 38% for severe asthma, and 62% for COPD exacerbation. Regarding the history: 67% were under long-term corticosteroid, 48% smokers, 29% received antibiotics, and all of them presented a clinical bronchospasm, and severity criteria for respiratory distress. Sixty percent of patients were hypoxic (SpO_2 _<92%). All patients received salbutamol inhalation, associated with inhaled anticholinergic agent in 71.4%. Intravenous glucocorticoid drug was dispensed in 71.4% and intravenous salbutamol in 23.8%. None of the asthma patients was intubated, five COPD patients (24.8%) were intubated. Twelve patients were admitted to the ICU (one with asthma and 11 with COPD). Comparison of clinical parameters between prehospital care and the emergency room shows a significant difference (*P *< 0.05) for respiratory rate (35.9 ± 7.48 vs. 24.95 ± 8.25) and pulse oxymetry (81.8 ± 15.8 vs. 96.4 ± 3.54).

## Conclusion

NIV through CPAP associated with nebulizations appears to provide benefit by reducing respiratory work (decreased respiratory rate) and improving alveolar ventilation (increased SpO_2_) in patients with asthma. However, in COPD patients, no improvement of symptoms has been observed.

